# The PlA1/A2 Polymorphism of Glycoprotein IIIa as a Risk Factor for Myocardial Infarction: A Meta-Analysis

**DOI:** 10.1371/journal.pone.0101518

**Published:** 2014-07-02

**Authors:** Christopher N. Floyd, Agnesa Mustafa, Albert Ferro

**Affiliations:** Department of Clinical Pharmacology, Cardiovascular Division, British Heart Foundation Centre of Research Excellence, King's College London, London, United Kingdom; University Heart Center Freiburg, Germany

## Abstract

**Background:**

The PlA2 polymorphism of glycoprotein IIIa (GPIIIa) has been previously identified as being associated with myocardial infarction (MI), but whether this represents a true association is entirely unclear due to differences in findings from different studies. We performed a meta-analysis to evaluate whether this polymorphism is a risk factor for MI.

**Methods:**

Electronic databases (MEDLINE and EMBASE) were searched for all articles evaluating genetic polymorphisms of GPIIIa. For studies where acute coronary events were recorded in association with genetic analysis, pooled odds ratios (ORs) were calculated using fixed-effects and random-effects models. The primary outcome measure was MI, and a secondary analysis was also performed for acute coronary syndromes (ACS) more generally.

**Findings:**

57 studies were eligible for statistical analysis and included 17,911 cases and 24,584 controls. Carriage of the PlA2 allele was significantly associated with MI (*n* = 40,692; OR 1.077, 95% CI 1.024–1.132; p = 0.004) but with significant publication bias (p = 0.040). The degree of association with MI increased with decreasing age of subjects (≤45 years old: *n* = 9,547; OR 1.205, 95% CI 1.067–1.360; p = 0.003) and with adjustment of data for conventional cardiovascular risk factors (*n* = 12,001; OR 1.240, 95% CI 1.117–1.376; p<0.001). There was a low probability of publication bias for these subgroup analyses (all p<0.05).

**Conclusions:**

The presence of significant publication bias makes it unclear whether the association between carriage of the PlA2 allele and MI is true for the total population studied. However for younger subjects, the relative absence of conventional cardiovascular risk factors results in a significant association between carriage of the PlA2 allele and MI.

## Introduction

The fibrinogen receptor is the most abundant integrin on the platelet surface [1], and through binding a combination of fibrinogen, von Willebrand factor and fibronectin, its main function is to terminate haemorrhage following vascular injury. Despite this important physiological function, it also plays a pathological role when stimulated excessively or inappropriately, and is a principal mediator of acute coronary thrombosis [2].

The glycoprotein IIIa (GPIIIa) subunit of the fibrinogen receptor has a number of stable allelic variants resulting from single amino acid substitutions [3], of which the PlA1/A2 diallelic antigen system (involving a leucine-proline polymorphism at position 33, which alters the protein conformation and spatial orientation of the ligand-binding region) has been associated with an increased prevalence of cardiovascular disease. In 1996, Weiss *et al* reported an association between carriage of the PlA2 allele and cardiovascular disease, with the association most marked in subjects with unstable angina or myocardial infarction (MI) under 60 years of age (Odds ratio (OR) 6.2, 95% confidence interval (CI) 1.8–22.4; p = 0.002) [4]. The subsequent literature has demonstrated marked inter-study variation in the level – and indeed presence – of such an association, which in part reflects generally underpowered investigations of an allele with a frequency in Caucasian populations of approximately 15 per 100 [5], falling to 1 per 100 in Oriental populations [6].

A number of meta-analyses have investigated the association between carriage of the PlA2 allele and cardiovascular disease. In 2001 Di Castelnuovo *et al* identified a significant association between PlA2 carriage and coronary artery disease (*n* = 17,049; OR 1.10, 95% CI 1.03–1.18), but found no significant association with MI (*n* = 11,628; OR 1.09, 95% CI 0.97–1.22) [7]. A subsequent meta-analysis also found no association with either MI (*n* = 30,630; per-allele RR 1.02, 95% CI 0.96–1.07) or coronary stenosis (*n* = 12,741; per-allele RR 1.04, 95% CI 0.97–1.13) [8].

The identification of a contribution by a single gene polymorphism to a multifactorial, polygenic pathological process is challenging and requires a very large sample size [9]. Here we present the largest meta-analysis to date, involving 42,495 subjects, to address the question of whether carriage of the PlA2 polymorphism is an independent risk factor for acute coronary events.

## Methods

### Search strategy and selection criteria

Electronic databases (MEDLINE and EMBASE) were searched without language restriction up until 1^st^ April 2013 for all articles evaluating genetic polymorphisms in the platelet GPIIIa receptor. The Medical Subject Headings terms used for the primary search related to genetics (e.g. ‘gene’, ‘polymorphism’, ‘mutation’ and ‘genotype’) in combination with glycoprotein IIIa (e.g. ‘glycoprotein IIIa’, ‘GP IIIa’ and ‘integrin beta 3’). Following removal of duplicates, a total of 2,288 articles were identified in the primary search. To further encompass all relevant literature, a secondary search of the references from reviews and included studies was performed.

All articles that investigated the association between carriage of the PlA2 polymorphism and acute coronary syndromes (ACS) were considered potentially eligible for inclusion and, based on analysis of title and abstract, 114 potentially suitable articles were identified. For inclusion into the meta-analysis, studies must have reported the distribution of the PlA1/A2 genotype in relation to the prevalence of a coronary event, either as raw data or calculated ORs. Both case-control and cohort studies were considered, with familial-based studies and studies without an English translation excluded.

**Figure 1:**
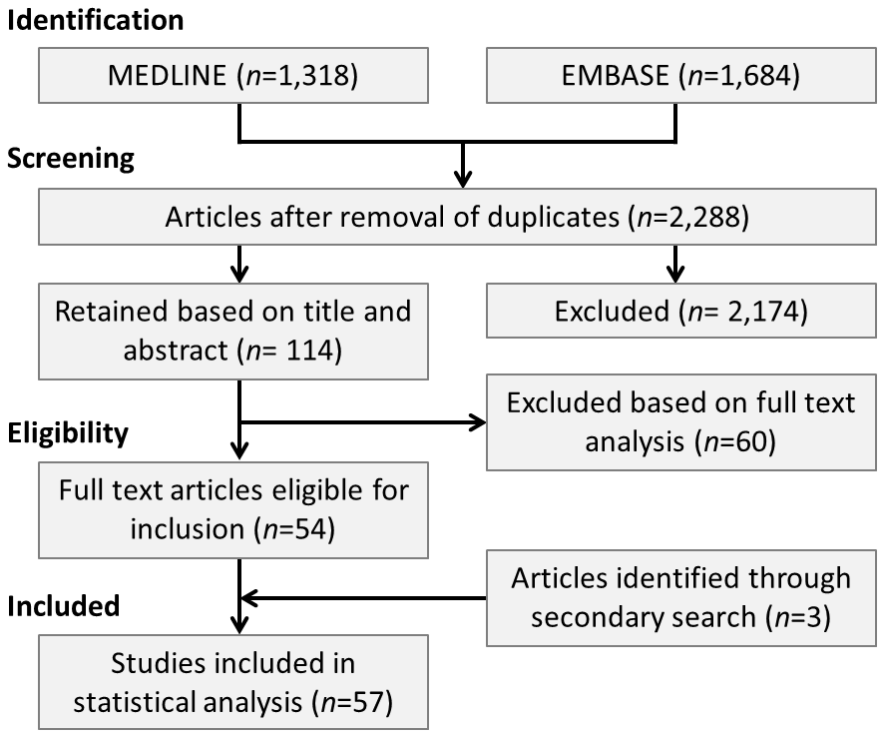
Summary of strategy used to identify studies suitable for analysis.

A number of studies were not suitable for inclusion based on the following reasons: 12 studies were unavailable in English [10]–[21], three studies were familial-based [22]–[24], seven studies reported data that duplicated or overlapped with larger studies that were eligible for inclusion [25]–[31], 26 studies did not contain suitable outcome data [32]–[57], and in 11 studies all subjects had a coronary event and so there was no control population [58]–[68]. Following the addition of three studies identified in the secondary search [69]–[71], a total of 57 studies met the inclusion criteria for statistical analysis (Figure 1).

### Data extraction

Data were extracted from each study according to a predefined protocol: study design, number of cases/controls, geographic location and/or ethnicity, sex and clinical outcome. The primary outcome measure was MI with a secondary outcome being ACS more generally. For the secondary outcome measure, data on the incidence of ACS were included preferentially where MI incidence was also reported.

Ethnic group was recorded where explicitly stated within a study. Where genotype information was reported for >1 sub-population as defined by geographic region or ethnic origin, each sub-population was considered separately in the analyses [9]. This was the case for studies by Herrmann *et al* and Kekomaki *et al* [72], [73].

Where data adjusted for age, gender, ethnicity and cardiovascular risk factors were available, these were analysed in preference to raw data. Similarly, in studies which had more than one control group, subjects with coronary artery disease were used in preference to healthy subjects as controls.

### Statistical analysis

Data were analysed using Comprehensive Meta-analysis software, version 2 (Biostat, USA). Pooled ORs were calculated using fixed- and random-effects models, along with the 95% CI to measure the strength of association. Fixed-effects summary ORs were calculated using the Mantel-Haenszel method [74], and the DerSimonian method was used to calculate random-effects summary Ors [75]. For data where more than one outcome was reported, combined effects were calculated as necessary [76]. Pooled ORs presented in the results were calculated using the fixed-effects model unless otherwise stated.

Tests for heterogeneity were performed for each meta-analysis, with significance set at p<0.05 [77]. I^2^ was also calculated for each analysis, where ≥50% may represent substantial heterogeneity [78]. For assessment of publication bias, we utilised a funnel plot and Egger's regression asymmetry test [79]. In addition, the effect of individual studies on the summary OR was evaluated by re-estimating and plotting the summary OR in the absence of each study.

## Results

**Figure 2:**
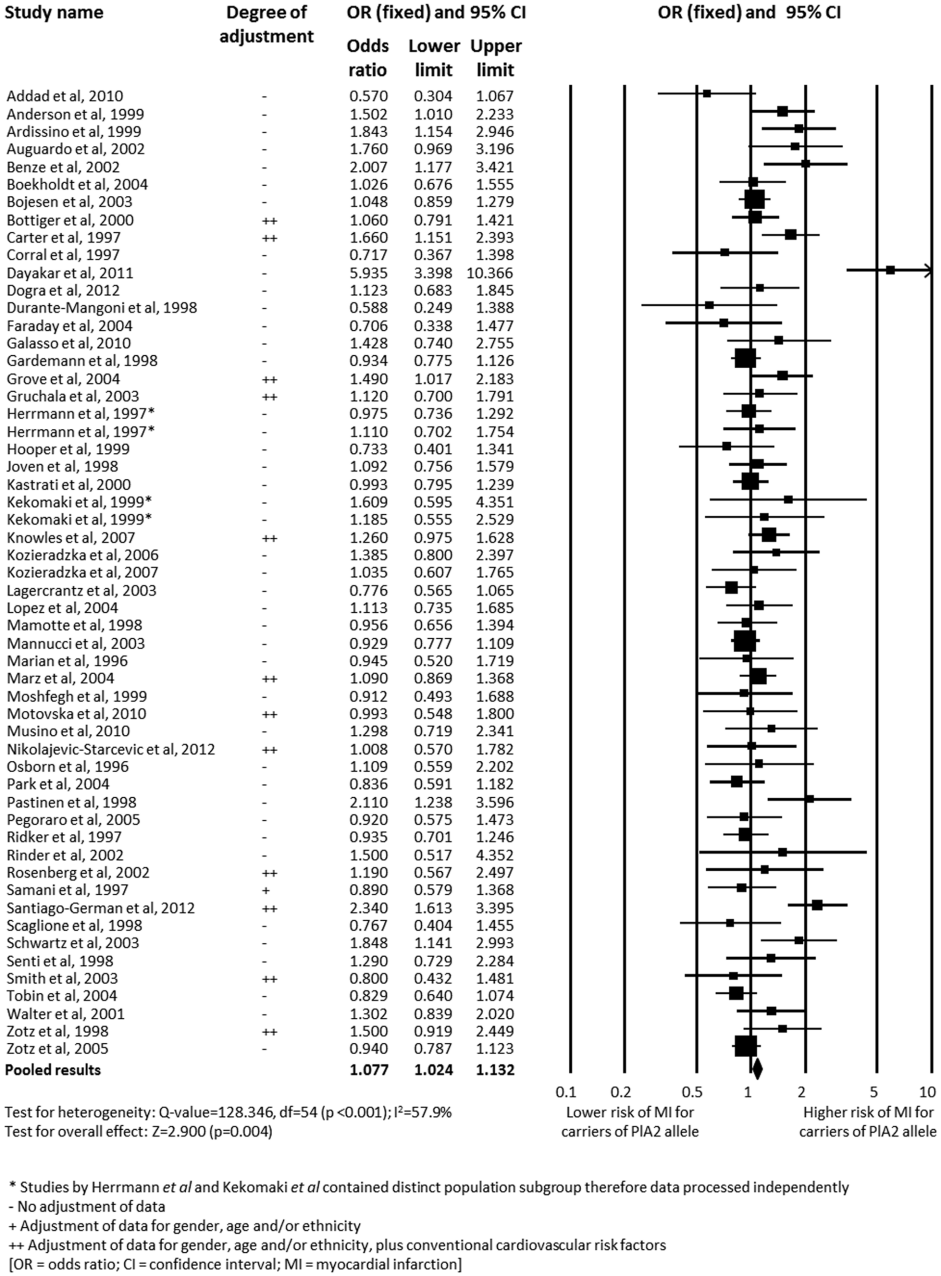
Analysis of the association between carriage of the PlA2 allele and myocardial infarction. Analysis is of the PlA1/A1 versus PlA1/A2+PlA2/A2 genotype.

We identified 53 studies with the endpoint of myocardial infarction, comprising a total of 16,863 cases and 23,829 controls. Pooled OR for the association of carriage of the PlA2 allele (PlA1/A2+PlA2/A2 versus PlA1/A1 genotype) with MI was significant at 1.077 (95% CI 1.024–1.132; p = 0.004) (Figure 2) [70]–[73], [80]–[128]. Significant heterogeneity was observed (I^2^ = 57.9%; p<0.001), and analysis using the random-effects model increased the degree of association (OR 1.132, 95% CI 1.039–1.232; p = 0.004). The addition of three further studies to assess the association with ACS more generally yielded an OR of 1.074 (*n* = 42,426; 95% CI 1.023–1.127; p = 0.004) (Table 1) [4], [69], [129].

**Table 1:** Association between carriage of the PlA2 polymorphism and acute coronary events.

	Number of studies	Number of cases/controls	Pooled OR* (95% CI)	Association (p value)	I^2^ (%)
**Primary analysis:**					
MI	53	16,863/23,829	1.077	0.004	57.9
	[70]–[73], [80]–[128]		(1.024–1.132)		

**Secondary and subgroup analyses:**					
ACS	56	17,887/24,539	1.074	0.004	59.3
	[4], [69]–[73], [80]–[129]		(1.023–1.127)		
MI (adjusted data)	13	6,188/5,813	1.240	<0.001	47.6
	[87], [88], [96], [97], [101], [109], [110], [112], [119]–[121], [125], [127]		(1.117–1.376)		
PlA1/A1 vs PlA2/A2	31	7,245/16,591	1.023	0.287	40.8
	[70]–[72], [81], [82], [86]–[91], [95], [98]–[100], [102]–[104], [106]–[108], [111]–[114], [116], [117], [120], [122], [124], [128]		(0.877–1.192)		

*OR (odds ratio) calculated using fixed-effects model for carriage of the PlA2 allele vs PlA1 homozygous subjects.

[MI =  myocardial infarction; ACS =  acute coronary syndrome].

**Figure 3:**
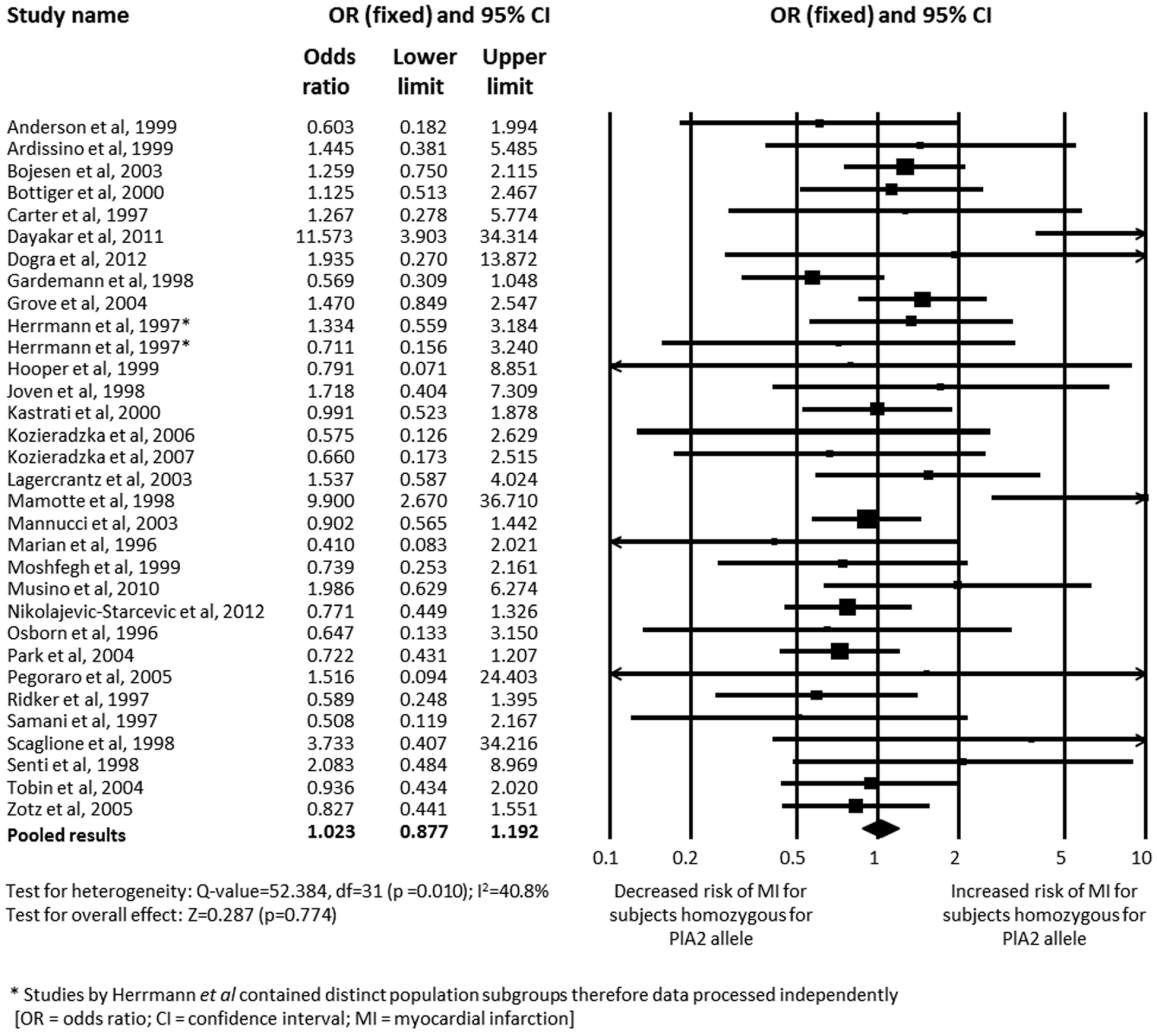
Analysis of the association with myocardial infarction between subjects homozygous for the PlA1 allele and those homozygous for the PlA2 allele.

Analysis of the association between carriage of the PlA2 allele and MI using data adjusted for age, sex, ethnicity and cardiovascular risk factors demonstrated an increased level of association (*n* = 12,001; OR 1.240, 95% CI 1.117–1.376; p<0.001) [87], [88], [96], [97], [101], [109], [110], [112], [119]–[121], [125], [127]. Further subgroup analysis based on comparison of the PlA1/A1 versus PlA2/A2 genotype failed to show a significant association (*n* = 23,836; OR 1.023, 95% CI 0.877–1.192; p = 0.774) [70]–[72], [81], [82], [86]–[91], [95], [98]–[100], [102]–[104], [106]–[108], [111]–[114], [116], [117], [120], [122], [124], [128] (Figure 3); however, within this analysis, the number of subjects with the PlA2/A2 genotype was small, consisting of 333 cases and 1,504 controls.

### Subgroup analyses based on subject demographics

Data were available to calculate pooled ORs based on subject ethnicity, sex and age (Table 2). Analysis based on ethnicity was limited to Caucasians as the majority of studies did not explicitly state the ethnicity of participants. A non-significant pooled OR for the association of PlA2 carriage with MI was observed for the 11 available studies (*n* = 10,585; OR 1.050, 95% CI 0.962–1.146; p = 0.272) [71], [81], [88], [95]–[97], [103], [109], [112], [127], [128].

**Table 2:** Subgroup analyses of the association between carriage of the PlA2 allele and myocardial infarction by subject demographics.

	Number of studies	Number of cases/controls	Pooled OR* (95% CI)	Association (p value)	I^2^ (%)
Caucasian	11	5,047/5,538	1.050	0.272	51.9
	[71], [81], [88], [95]–[97], [103], [109], [112], [127], [128]		(0.962–1.146)		
Male	11	2,715/5,971	1.145	0.024	39.0
	[72], [84]–[86], [97], [99], [114], [115], [120], [123], [127]		(1.018–1.288)		
Female	2	249/4,988	0.961	0.801	0.0
	[86], [120]		(0.703–1.312)		
**Subgroup analyses based on age of first event:**					
Age ≤65 years old†	23	5,216/10,952	1.101	0.029	57.4
	[72], [82], [84], [86], [91], [97], [99], [106], [107], [110], [111], [114], [116], [119], [121]–[123], [140]		(1.010–1.201)		
Age ≤55 years old†	18	3,744/6,017	1.144	0.007	58.5
	[72], [82], [84], [86], [91], [97], [99], [106], [107], [110], [111], [114], [116], [119], [121]–[123], [140]		(1.037–1.261)		
Age ≤45 years old†	11	3,675/5,872	1.205	0.003	70.3
	[72], [82], [84], [86], [91], [97], [107], [116], [121]–[123]		(1.067–1.360)		
First MI§	15	5,011/13,338	1.131	0.006	81.2
	[82], [84], [86], [87], [90], [101], [104], [107], [111], [117], [119]–[122], [124], [125]		(1.036–1.234)		

*OR (odds ratio) calculated using fixed-effects model for carriage of the PlA2 allele vs PlA1 homozygous subjects.

<?ENTCHAR dagger?> Age defined as age of onset of event.

<?ENTCHAR sect?> Event recorded as the first MI experienced by the subject.

[MI =  myocardial infarction].

The association between PlA2 carriage and MI in male subjects was observed to be consistent with the primary analysis (*n* = 8,686; OR 1.145, 95% CI 1.018–1.288; p = 0.024) [72], [84]–[86], [97], [99], [114], [115], [120], [123], [127]. Only two studies provided data for female subjects, resulting in no significant association observed in these (*n* = 5,237; OR 0.961, 95% CI 0.703–1.312; p = 0.801) [86], [120].

**Figure 4:**
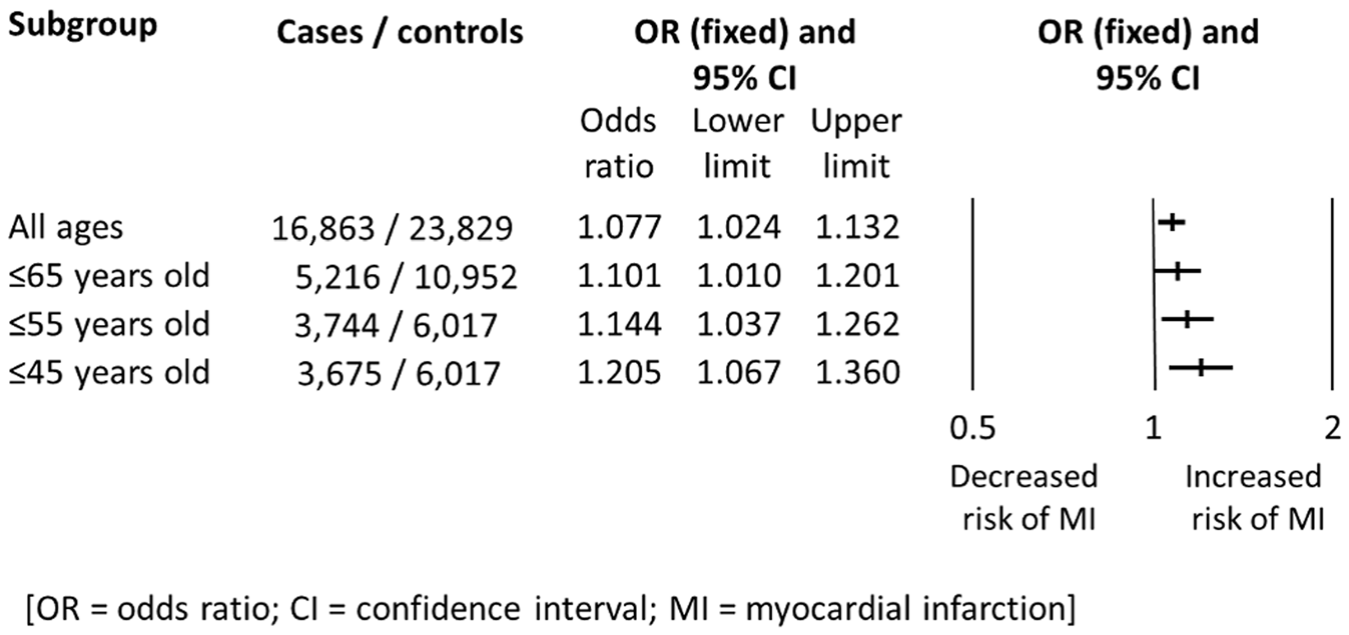
Summary of subgroup analyses based on age at first event. Analysis is of the PlA1/A1 versus PlA1/A2+PlA2/A2 genotype.

Subgroup analyses based on subject age at first MI demonstrated an increased level of association with decreasing age (Figure 4). For subjects ≤45 years old, carriage of the PlA2 allele produced a pooled OR for MI of 1.205 (*n* = 9,547; 95% CI 1.067–1.360; p = 0.003) with significant heterogeneity (I^2^ = 70.3%, p<0.001), and analysis using the random-effects model again increased the level of association (OR 1.356, 95% CI 1.044–1.762; p = 0.022) [72], [82], [84], [86], [91], [97], [107], [116], [121]–[123]. Significant heterogeneity and an increased level of association as compared with the total population were also observed using the random-effects model for the ≤55 and ≤65 year-old subgroups.

In 15 of the studies identified, the recorded cardiovascular event was a ‘first event’ for the participant. Analysis of these studies revealed an association between carriage of the PlA2 allele and MI that was stronger than that seen in the primary analysis (*n* = 18,349; OR 1.131, 95% CI 1.036–1.234; p = 0.006) [82], [84], [86], [87], [90], [101], [104], [107], [111], [117], [119]–[122], [124], [125].

### Subgroup analyses based on study characteristics

**Figure 5:**
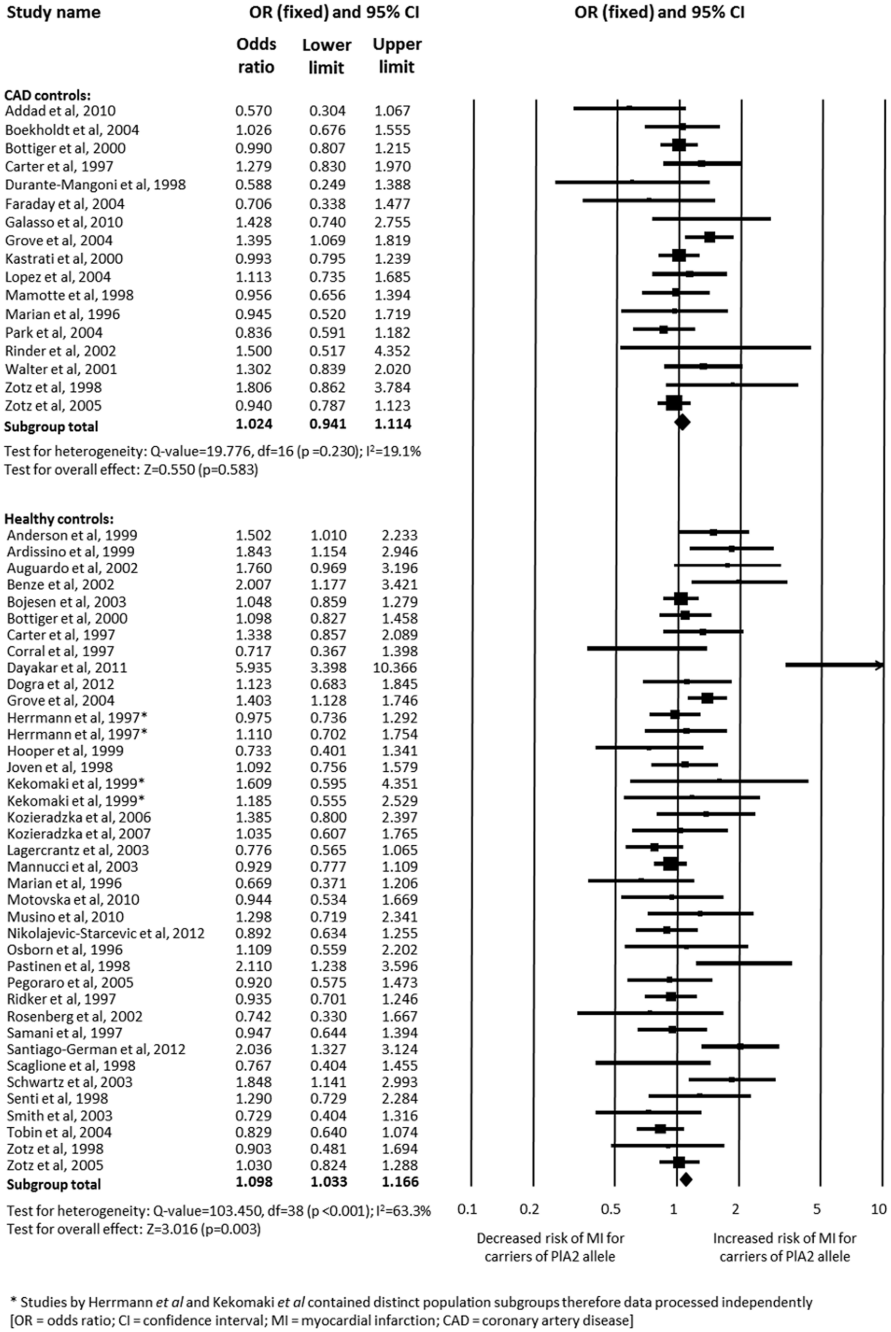
Analysis of the association between carriage of the PlA2 allele and myocardial infarction based on the use of healthy controls or controls with known coronary artery disease. Analysis is of the PlA1/A1 versus PlA1/A2+PlA2/A2 genotype.

As described within the Methods, adjusted data were analysed in preference to raw data wherever possible in order to distinguish the true effect of the PlA2 allele against a background of conventional cardiovascular risk factors. Similarly, in studies where more than one control population was included, the group with coronary artery disease was selected in preference to healthy subjects as the control group. These data are all displayed in Table 3. Subgroup analysis of crude data using only healthy controls found an increased association between PlA2 carriage and MI compared to the primary analysis (*n* = 29,907; OR 1.098, 95% CI 1.033–1.166; p = 0.003) [71]–[73], [81]–[84], [86]–[91], [96], [98], [99], [102]–[104], [107], [108], [110]–[113], [115]–[117], [119]–[125], [127], [128], whereas analysis of raw data using controls with known coronary artery disease found no significant association (*n* = 11,819; 95% CI 0.941–1.114; p = 0.583) (Figure 5) [80], [85], [87], [88], [92]–[94], [96], [100], [105], [106], [108], [114], [118], [126]–[128].

**Table 3:** Subgroup analyses of the association of carriage of the PlA2 allele and myocardial infarction by study characteristics.

	Number of studies	Number of cases/controls	Pooled OR* (95% CI)	Association (p value)	I^2^ (%)
**Subgroup analyses based on control population:**					
Controls with CAD	17	10,458/19,449	1.024	0.583	19.1
	[80], [85], [87], [88], [92]–[94], [96], [100], [105], [106], [108], [114], [118], [126]–[128]		(0.941–1.114)		
Healthy controls	39	4,883/7,016	1.098	0.003	63.3
	[70]–[73], [81]–[84], [87]–[91], [96], [98], [99], [101]–[104], [107], [108], [110], [111], [113], [115]–[117], [119]–[124], [127], [128]		(1.033–1.166)		
**Subgroup analyses based on study design:**					
Cohort	17	6,192/12,840	0.996	0.926	0.0
	[80], [85], [86], [92]–[95], [97], [100], [105], [106], [109], [112], [114], [118], [125], [126]		(0.917–1.082)		
Case-control	36	10,671/10,989	1.126	<0.001	66.7
	[70]–[73], [81]–[84], [87]–[91], [96], [98], [99], [101]–[104], [107], [108], [110], [111], [113], [115]–[117], [119]–[124], [127], [128]		(1.057–1.198)		
**Subgroup analyses based on number of cases:**					
<250 cases	37	6,462/3,683	1.240	0.006	59.2
	[70], [72], [73], [80]–[83], [85], [88]–[94], [97], [98], [102], [103], [105], [108], [110], [111], [113]–[116], [118]–[127], [130]		(1.139–1.350)		
≥250 cases	17	10,401/20,146	0.999	0.864	25.3
	[71], [72], [84], [86], [87], [95], [96], [99]–[101], [104], [106], [107], [109], [112], [117], [128]		(0.935–1.084)		

*OR (odds ratio) calculated using fixed-effects model for carriage of the PlA2 allele vs PlA1 homozygous subjects.

[CAD =  coronary artery disease].

**Figure 6:**
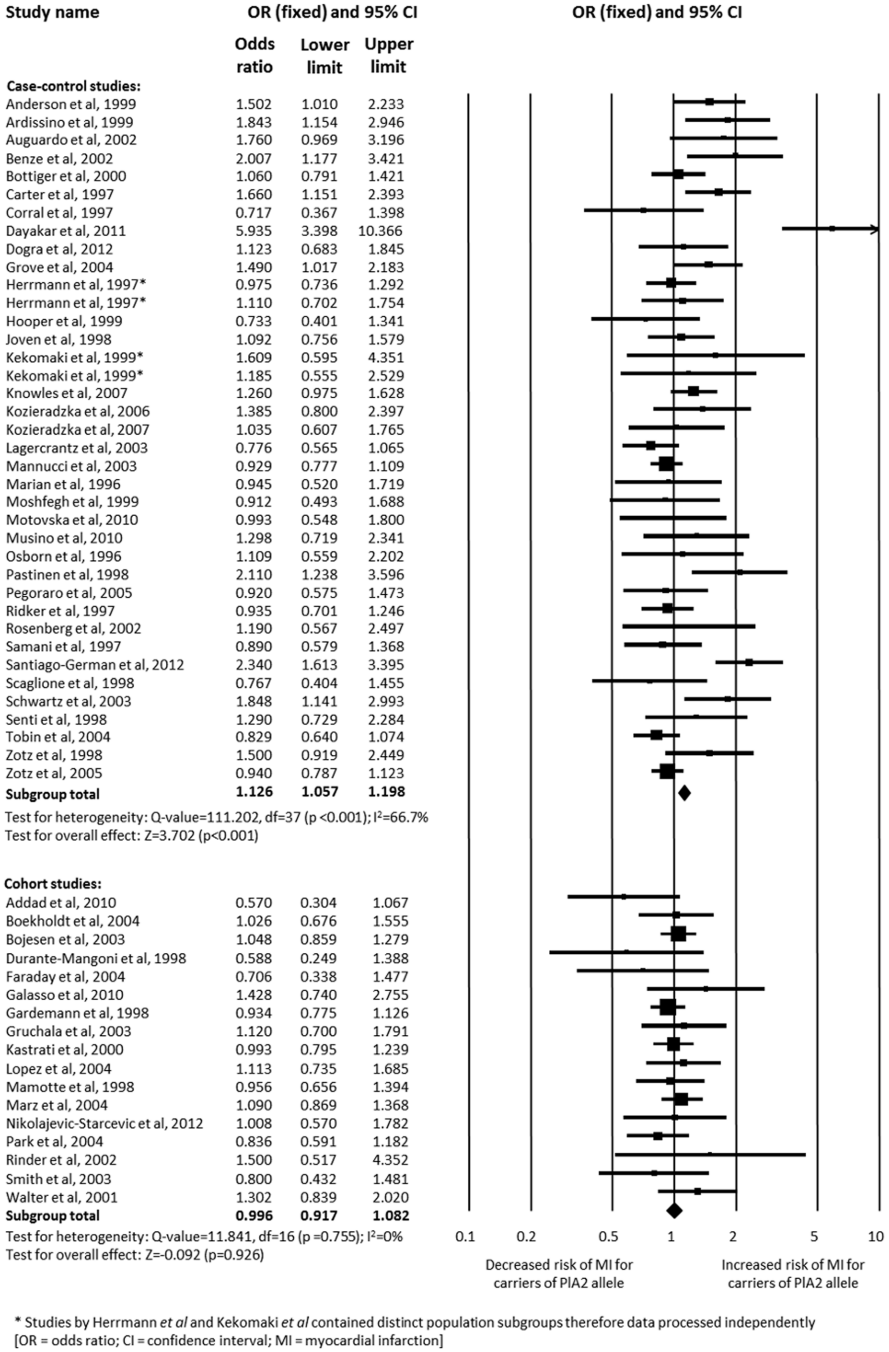
Analysis of the association between carriage of the PlA2 allele and myocardial infarction based on study design. Analysis is of the PlA1/A1 versus PlA1/A2+PlA2/A2 genotype.

Study design was found to influence the degree of association. Cohort studies did not demonstrate an association between carriage of the PlA2 allele and MI (*n* = 19,032; OR 0.996, 95% CI 0.917–1.082; p = 0.926) [80], [85], [86], [92]–[95], [97], [100], [105], [106], [109], [112], [114], [118], [125], [126], whereas case-control studies did (*n* = 21,660; OR 1.126, 95% CI 1.057–1.198; p<0.001) (Figure 6) [70]–[73], [81]–[84], [87]–[91], [96], [98], [99], [101]–[104], [107], [108], [110], [111], [113], [115]–[117], [119]–[124], [127], [128]. Studies with <250 cases showed a significant association (*n* = 10,145; OR 1.240, 95% CI 1.139–1.350; p = 0.006) [70], [72], [73], [80]–[83], [85], [88]–[94], [97], [98], [102], [103], [105], [108], [110], [111], [113]–[116], [118]–[127], [130], whereas studies with ≥250 cases did not (*n* = 30,547; OR 0.999, 95% CI 0.935–1.084; p = 0.864) [71], [72], [84], [86], [87], [95], [96], [99]–[101], [104], [106], [107], [109], [112], [117], [128]. Eleven out of 17 studies with ≥250 cases were case-control studies [71], [72], [84], [87], [99], [101], [104], [107], [117], [120], [128].

### Publication bias

**Figure 7:**
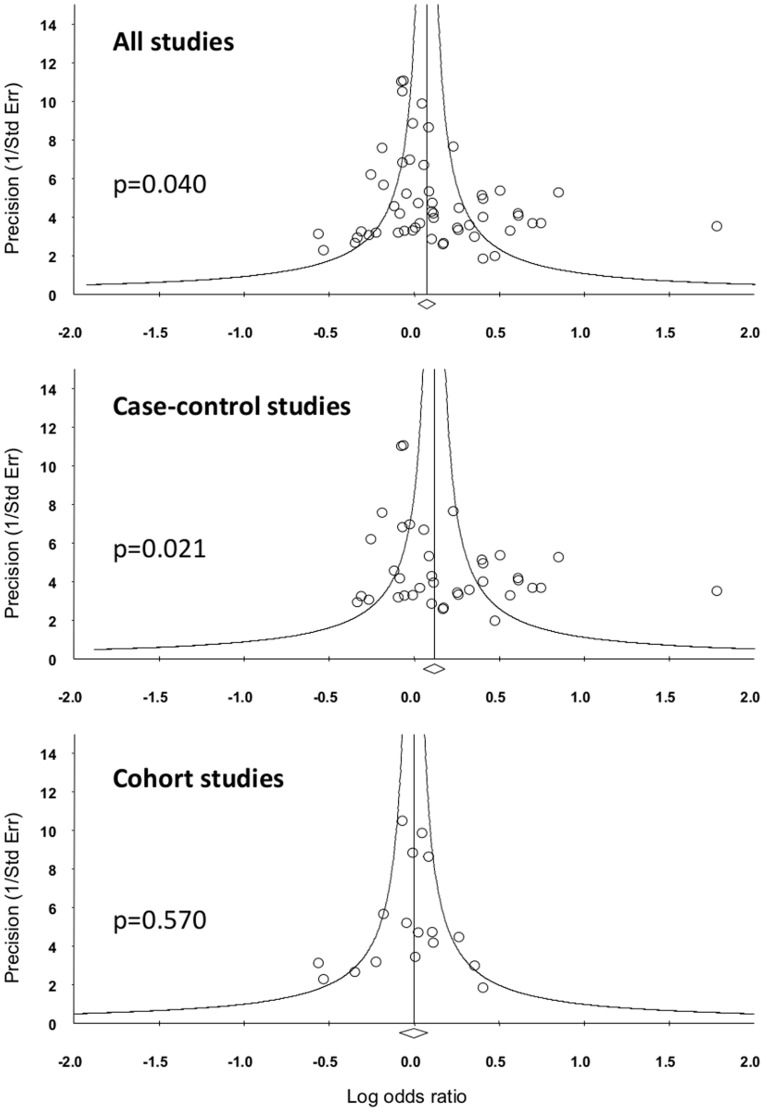
Funnel plots to assess publication bias. For each study, the log odds ratio is shown against study precision. The open diamond below the x-axis indicates the pooled odds ratio. p-values are reported for Egger's regression intercept, where p>0.05 suggests a low probability of publication bias.

Publication bias was assessed by plotting funnel plots and calculation of Egger's regression intercept. The funnel plot for the primary analysis was asymmetric with a significant Egger's regression intercept (p = 0.040), suggesting the likelihood of publication bias skewed towards studies that favour an association between carriage of the PlA2 allele and MI (Figure 7). This bias was independent of study size (<250 cases, p = 0.264; ≥250 cases, p = 0. 088), but dependent on study design (case-control studies, p = 0.021; cohort studies, p = 0.570). Further analysis of case-control studies suggested that this bias was not associated with study size (<250 cases, p = 0.157; ≥250 cases, p = 0.066).Significant bias was not present for the subgroup analyses based on age (≤65, p = 0.161; ≤55, p = 0.191; ≤45, p = 0.192).

## Discussion

The data presented here appears to demonstrate an increased risk of MI in carriers of the PA2 allele, with an association that becomes stronger as age decreases. Significant publication bias identified in the primary analysis makes it unclear whether the association is true for the totality of the population studied. This bias is primarily driven by case-control studies and surprisingly independent of study size given that smaller studies are generally more prone to this effect. The presence of bias is consistent with the general findings of the two previous meta-analyses [7], [8]. However despite these concerns, there remains a clear age effect with the association between carriage of the PlA2 allele and MI most evident for younger age cohorts.

The observed age-skewed risk profile may be explained by a relative (rather than absolute) decrease in the influence of genetic factors with age, given that the prevalence of conventional cardiovascular risk factors increases with age [131]. This hypothesis is supported by the increased association between carriage of the PlA2 allele and MI observed in the subgroup analysis using data adjusted for these risk factors. Similarly, subgroup analysis using unadjusted data and controls with coronary artery disease resulted in no significant association seen, and this is likely to be explained again by the dilution effect caused by the coexistence of conventional risk factors.

### Aetiology of increased risk

It is unclear as to the mechanism by which carriage of PlA2 predisposes to increased cardiovascular morbidity and mortality. The aetiology of cardiovascular risk is not monogenic, but a complex polygenic interaction with environmental factors [131], with each additional factor contributing in an additive and sometimes in a synergistic manner [132]. This contribution can be clearly demonstrated in the increased risk that smoking adds to carriage of the PlA2 allele when compared to non-smoking PlA1 homozygotes [82], [96], [105], [123]. It has been suggested that smoking and PlA2 increase cardiovascular risk via interacting mechanisms [133], but given the strong association of smoking with premature myocardial infarction [134], further data are required to test the strength of this association.

There remains the possibility of unidentified linkage disequilibrium with genes modulating other conventional cardiac risk factors, with elevated plasma lipids being previously linked to carriage of the PlA2 allele [133]. This hypothesis is however not supported by the data analysed within the present meta-analysis, with only one study finding higher triglycerides in carriers of the PlA2 allele [80] and conflicting reports on the levels of lipoprotein(a) [86], [99]. Interestingly, Grove *et al* found that the association between carriage of the PlA2 allele and MI decreased as cholesterol levels increased, suggesting once again that the true effect of the PlA2 allele may be diluted and hence concealed by the concomitant presence of conventional risk factors [96].

The proximity of the PlA1/A2 epitope to the ligand binding site of GPIIIa has led investigators to consider how the single amino acid substitution of proline for leucine may affect the cycle of ligand association and dissociation with the fibrinogen receptor. Studies have been inconclusive, with no difference observed in static systems but an enhancement of binding and outside-in signalling seen in cell culture under conditions of shear stress, thus potentially resulting in circulating platelets having a higher basal level of activation [2]. Similarly to plasma lipids, plasma fibrinogen concentration has been suggested as a potential modulator of the increased risk secondary to carriage of the PlA2 allele, but as with the lipid hypothesis, studies included in this meta-analysis do not support this association. Three studies reported a higher levels of fibrinogen in certain subgroups of individuals carrying the PlA2 allele [85], [86], [121] and two studies reported higher fibrinogen levels in PlA1 homozygous subjects [124], [125].

Resistance to aspirin has been suggested as another potential mechanism by which carriage of the PlA2 allele may cause increased cardiovascular risk [135]. However, a recent large meta-analysis has suggested that this is not the case [136]. There is however significant inter-study heterogeneity, and the need for further studies in this regard remains.

A final avenue of investigation has been whether the PlA1/A2 antigens affect the degree or morphology of atherosclerosis. Carotid plaque morphology was examined by magnetic resonance imaging in 1,202 participants in the atherosclerotic risk in communities (ARIC) study [46]. Subjects who carried the PlA2 allele were found to have plaques with thinner fibrous caps, and these thinner caps represent the major precursor lesion for ACS [137]. However, this study was limited by a low frequency of the minor allele and technical constraints resulting in plaque morphology being assessed only in individuals with thick arterial walls.

### Study limitations

A potential limitation of this meta-analysis is the presence of a mortality bias that may attenuate or entirely obscure any true association. Almost a third of individuals with a first major coronary event die out-of-hospital [138], and are not accounted for in the predominantly retrospective data presented in this meta-analysis. In fact, in most studies the subject must have survived a cardiac event for a number of months or even years to be available for inclusion. If carriage of the PlA2 allele is more likely to result in an immediately fatal cardiac event then the association would be attenuated, with the opposite effect observed if carriage of the PlA1 allele results in increased early mortality.

The potential presence of a mortality bias can be investigated by considering post-mortem data from out-of-hospital deaths. The Helsinki Sudden Death Study considered 700 white Finnish males (aged 33–70 years old) who had sudden, unexpected out-of-hospital deaths [139]. Carriage of the PlA2 allele was significant associated with acute coronary thrombosis in those diagnosed with sudden cardiac death (OR 3.4; 95% CI 1.5–6.3), with an increased association observed in those <60 years old (OR 4.6; 95% CI 2.0–11.2). These results do suggest the presence of a mortality bias, but the data are not directly comparable to those presented within the present meta-analysis. Nevertheless, they do indicate that carriage of the PlA2 has an increased association with platelet-mediated thrombotic cardiac events, and are therefore consistent with the increased OR seen for the clinical outcome of MI or indeed ACS.

## Conclusions

In conclusion, carriage of the PlA2 allele is a significant risk factor for the development of acute coronary events in younger subjects, with data representing the total population subject to significant publication bias. This age-skewed risk profile appears to be the result of the relative impact of the polymorphism becoming attenuated as conventional cardiovascular risk factors develop with advancing age. The precise mechanism by which carriage of the PlA2 allele leads to increased cardiovascular risk remains unclear, however this should not impair its potential utility in risk stratification of younger subjects with modifiable risk factors.

## Supporting Information

Checklist S1 **PRISMA Checklist.**
(DOC)
